# Healthcare can’t stop evolving: innovation as the catalyst for unleashing the managerial potential of value-based healthcare by stimulating intangible assets and enhancing organizational resilience

**DOI:** 10.3389/fpsyg.2024.1438029

**Published:** 2024-09-19

**Authors:** João Francisco Pollo Gaspary, Vinícius Jaques Gerhardt, Claudia de Freitas Michelin, Luis Felipe Dias Lopes, Carmen Brum Rosa, Julio Cezar Mairesse Siluk

**Affiliations:** ^1^Postgraduate Program in Production Engineering, Federal University of Santa Maria, Santa Maria, Brazil; ^2^Postgraduate Program in Accounting Sciences, Federal University of Santa Maria, Santa Maria, Brazil; ^3^Postgraduate Program in Administration, Federal University of Santa Maria, Santa Maria, Brazil

**Keywords:** value-based health care, organizational resilience: intangible assets in healthcare, healthcare management, applied research, work break structure, patient outcomes measurement, healthcare innovation and quality

## Abstract

**Background:**

With increasing healthcare service utilization and the introduction of costly therapies, healthcare organizations are pressured to deliver cost-effective services within constrained budgets. Rising costs and the need for efficient healthcare delivery are major concerns for governments, insurers, and health plans.

**Objectives:**

It aims to understand the impact of these intangible assets on creating value and organizational resilience in healthcare, informing better practices and strategies for VBHC implementation.

**Methods:**

An applied research approach using the Work Breakdown Structure (WBS) methodology was adopted. The research was divided into seven interconnected Work Packages (WPs), each designed to investigate different aspects of the integration between VBHC and intangible assets, with a focus on enhancing organizational resilience through innovative health processes. Key methodologies included literature reviews and qualitative analyses, employing Open Innovation and Design Thinking.

**Results:**

The study revealed a dynamic interplay between VBHC, organizational resilience, and intangible assets. It showed that managerial effectiveness is influenced by direct patient outcomes and elements like intellectual capital and organizational reputation. Data integration from various Work Packages provided new insights into how intangible assets underpin VBHC strategies, proposing novel management approaches. Findings highlight the essential role of intangible assets in enhancing service delivery and fostering sustainable healthcare practices.

**Discussion:**

The study highlights a significant oversight in the integration of intangible assets within healthcare organizations, despite their crucial role in optimizing VBHC. It supports literature emphasizing the importance of intellectual capital and organizational culture in enhancing healthcare management efficiency and resilience. A paradigm shift in VBHC to include these assets is needed for building a more adaptable and sustainable healthcare system. This integration can lead to better clinical outcomes, patient satisfaction, and overall healthcare efficiency, aligning more closely with VBHC goals.

**Conclusion:**

Recognizing and effectively managing intangible assets are paramount for the successful implementation of VBHC and enhanced organizational resilience. Strategic integration of these assets into healthcare management practices can significantly improve patient outcomes and create a more sustainable, patient-centered, and resilient healthcare system. Future studies should develop methodologies for robust measurement and integration of these assets to fully realize the potential of VBHC.

## 1 Introduction

As the use of healthcare services increases and the introduction of costly new therapies continues to pose challenges, healthcare organizations are under significant pressure to deliver services within sustainable budgets. Governments, insurers, and health plans alike are deeply concerned about the escalating costs and the efficiency of healthcare delivery. In this complex landscape, Value-Based Health Care (VBHC) has emerged as a crucial framework, recognized for its potential to manage healthcare effectively by focusing on maximizing patient outcomes relative to costs (Porter and Teisberg, 2006; Porter, 2010; Makdisse et al., 2020; van Staalduinen et al., 2022).

Historically, healthcare management has focused predominantly on tangible indicators for assessing performance. However, recent insights suggest a significant shift towards recognizing the importance of intangible assets in creating value in healthcare. According to Walraven et al. (2022), VBHC should prioritize patient-centric measures—such as treatment efficiency, safety, adherence, and satisfaction—which are inherently multidimensional and vary among individuals and across time (Riva and Pravettoni, 2016). This perspective aligns VBHC with the broader definition of health as described by the World Health Organization, emphasizing physical, mental, and social well-being, not merely the absence of disease (Riva and Pravettoni, 2016).

To address these multidimensional aspects of healthcare, Porter and Teisberg (2006) operationalized VBHC through a framework that includes organizing care into Integrated Practice Units (IPUs), measuring outcomes and costs for each patient, and transitioning to bundled payments, among other strategies (Porter and Teisberg, 2006). This approach, however, provided limited guidance on the implementation strategies appropriate for different healthcare settings, as noted by van Staalduinen et al. (2022).

The recognition of intangible assets such as organizational culture, intellectual capital, and patient satisfaction is increasingly acknowledged as crucial for enhancing the sustainability and effectiveness of healthcare management. Our study employs a comprehensive applied research approach using the Work Breakdown Structure (WBS) methodology, as described by the Project Management Institute (2021). This methodology divides the research into six interconnected Work Packages (WPs), each aimed at exploring and enhancing the integration of intangible assets within VBHC frameworks to promote a comprehensive and multidisciplinary perspective on healthcare management.

Each WP involves a detailed qualitative analysis to ensure the applicability of findings in clinical settings, with the overarching goal of developing a robust framework for integrating intangible assets effectively within VBHC. This systematic approach seeks to uncover updated managerial strategies that leverage these assets for enhanced healthcare delivery and organizational performance.

Through a rigorous methodology outlined in subsequent WPs, this research systematically maps, integrates, and analyzes intangible assets using extensive literature reviews and data integration techniques. The process involves mapping VBHC systems, identifying challenges in implementation, and exploring innovative solutions to enhance organizational resilience and effectiveness. By reevaluating and prioritizing these assets, our research aims to provide healthcare managers with actionable insights and a robust framework for implementing VBHC strategies that truly reflect the value of intangible assets in delivering quality care.

## 2 Methods

**Figure 1:**
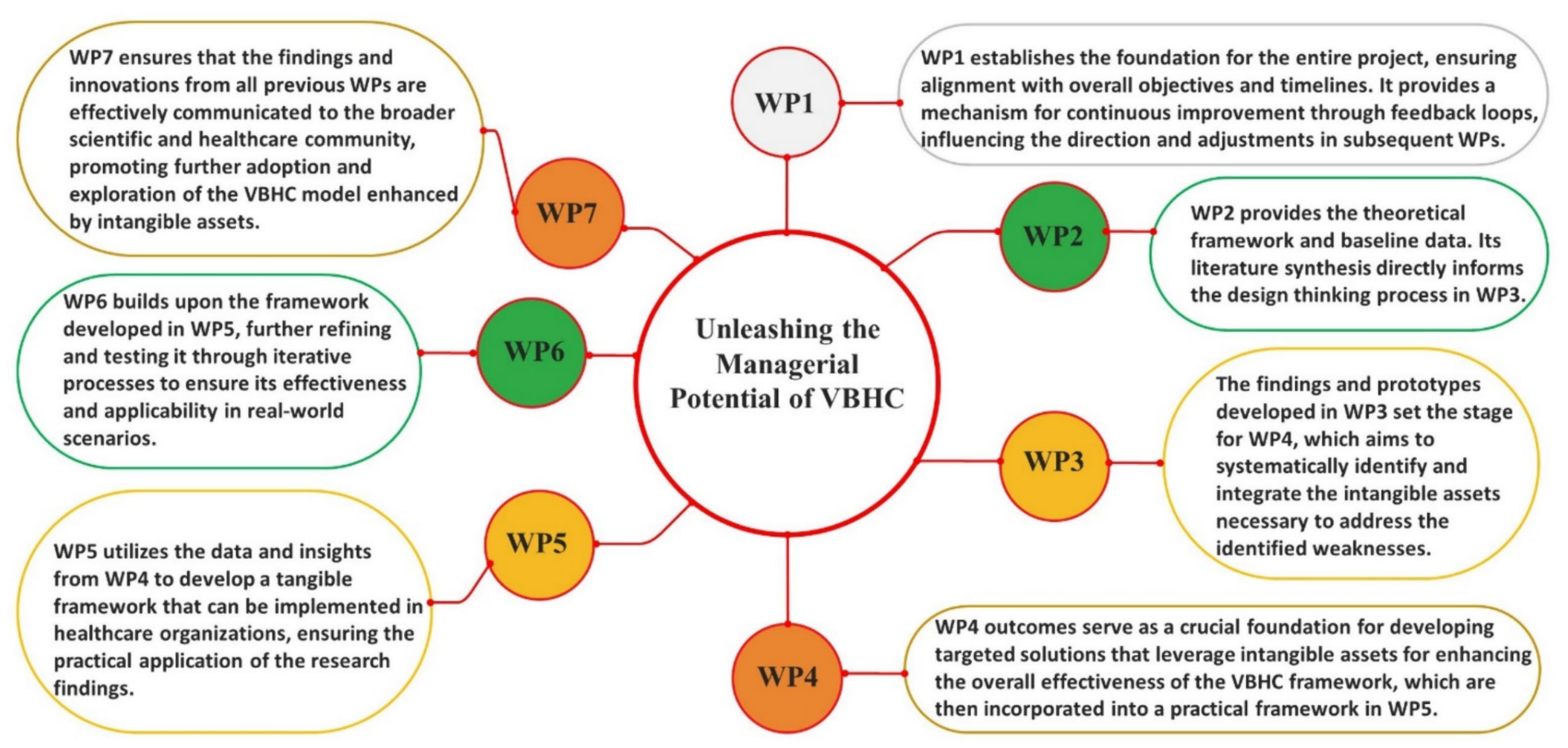
WPs connections.

In the preparation of this manuscript, OpenAI’s ChatGPT-4, a generative AI model, was utilized to aid in language refinement and drafting. The model’s responses were carefully reviewed and edited to ensure accuracy and alignment with the authors’ intent. To achieve the outlined objectives, applied research was conducted, structured using the Work Breakdown Structure (WBS) methodology as described by the Project Management Institute (2021). This management approach divided the research process into six smaller, well-defined, interconnected Work Packages (WP’s), each designed to explore the unleashing of the managerial potential of value-based healthcare: rescuing the duty of stimulating intangible assets, thereby promoting a comprehensive and multidisciplinary perspective. Each WP was followed by a detailed qualitative analysis of the results to ensure the relevance and applicability of the findings in a real scenario. The stages of the research are summarized in Figure 1, which presents the contributions, integrations, and methods of each WP.

The WBS methodology was chosen to add value to the innovation creation process, ensuring that ideas are presented step-by-step, demonstrating a commitment to high scientific research standards, similar to what was done by Gaspary et al. (2024) to present an innovative approach to treat cancer. This approach aligns with the principles discussed by Sargeant et al. (2010), which underscore the importance of embedding and tailoring such methodologies to specific organizational contexts to achieve their full potential. Compared to other methodologies, WBS offers a structured framework that allows for detailed exploration and systematic integration of various research components (Project Management Institute, 2021), making it particularly suitable for complex healthcare studies that require comprehensive analysis and iterative feedback (Oborn et al., 2013; Lim et al., 2019; Sood et al., 2021).

The specific objectives of WP1—Management and Supervision—were to ensure the correct functioning of the project and its realization based on the initially outlined objectives and timeline, to stimulate “team cohesion” using the Open Innovation methodology (Chesbrough, 2003), to review and adjust each WBS as the project progressed; to ensure clear mechanisms for feedback and communication between the WPs, and to evaluate the impact of new management perspectives developed. WP1 establishes the foundation for the entire project, ensuring that all other WPs are aligned with the overall objectives and timelines. It also provides a mechanism for continuous improvement through feedback loops, influencing the direction and adjustments in subsequent WPs.

The specific objective of WP2—Exploring VBHC, Health Innovation, and Organizational Resilience: A Literature Synthesis—was to generate an updated theoretical reference on VBHC, innovation in health and organizational resilience. This WP provides the theoretical framework and baseline data, which is crucial for the development of innovative strategies in WP3 and the systematic identification of intangible assets in WP4. WP2’s literature synthesis directly informs the design thinking process in WP3, ensuring that the strategies developed are grounded in current theoretical and empirical insights. For this purpose, an integrative literature review was conducted. To complete the first specific objective of this research—to perform an analysis on VBHC in the context of health service provision—and to extract the related literature for the organization of the theoretical framework of this research, a query was primarily developed on platforms such as Medline (via PubMed), Web of Science, Scielo, and Scopus.

Since this type of research can include scientific articles and documentary research that includes the Brazilian scenario, essential for the proper development of the structuring and planning of the business model. Articles relevant in a broad temporal scope were sought for most of the indexers used. Key terms related to the main words involving the specific research questions previously mentioned, and for each section of the theoretical framework that could return relevant articles, were defined. Table 1 specifies how the Integrative Literature Review occurred according to the criteria of Khan et al. (2011).

**Table 1:** Article selection criteria for a WP2 review for this study.

Topic	Article selection
VBHC	Database: LILACS; Medline; Web of Science; Scopus; SciELO; Google Scholar, Research Gate. Time Limit: Broad limit (2006–2024). Languages: English, Portuguese, or Spanish. Indexed Terms: “Value Based Healthcare” or “VBHC.”
Healthcare innovation	Database: LILACS; Medline; Scopus. Time Limit: Broad limit (2000–2024). Languages: English, Portuguese, or Spanish. Indexed Terms: “Innovation” and “healthcare.”
Organizational resilience	Database: LILACS; Medline; Scopus. Time Limit: Last 5 years (2019–2024). Languages: English, Portuguese, or Spanish. Indexed Terms: “Organizational resilience.”
Selection criteria	Inclusion: Peer-reviewed full articles. Exclusion: Commentary articles, editorials, conference abstracts. Eligibility: Articles discussing intangible assets, focusing on their identification, measurement, and impact on organizational performance.
Data extraction	Standardized data extraction form used to collect information: study characteristics (e.g., author, year of publication), methodology, key findings related to intangible assets, and implications for organizational performance and VBHC implementation.
Analysis methods	Thematic analysis conducted to identify common themes and patterns. data categorized into broader themes related to VBHC, healthcare innovation, and organizational resilience.
Risk of bias	Assessed methodological quality of included studies: clarity in defining intangible assets, rigor of measurement methods, strength of evidence linking intangible assets to organizational performance.
Synthesis of results	Narrative synthesis highlighting the variety of intangible assets recognized, their measurement, and impact. Thematic analysis to categorize intangible assets into broader themes.
Additional analyses	Subgroup analyses based on the type of intangible asset and sector (healthcare vs. non-healthcare).

Source: Adapted from Khan et al. (2011).

The specific objective of WP3—Design Thinking for Innovative Solutions in Organizational Resilience within VBHC—is to apply Design Thinking (Brown, 2008) and Open Innovation methodology (Chesbrough, 2003) to enhance organizational resilience by unleashing the managerial potential of value-based healthcare. Utilizing insights and theoretical frameworks from WP2, this Work Package will focus on ideating, prototyping, and testing innovative strategies that address and reinforce the management of intangible assets within healthcare organizations. This iterative process will identify potential weaknesses in the current VBHC management approach. Recognizing this, WP3 will set a new objective for WP4: to develop targeted solutions to address these identified weaknesses. The findings and prototypes developed in WP3 set the stage for WP4, which aims to systematically identify and integrate the intangible assets necessary to address the identified weaknesses.

The specific objective of WP4—Systematic Identification and Integration of Intangible Assets in VBHC—is to systematically identify and catalog all intangible assets mentioned in the scientific literature, thereby generating a selection of Fundamental Points of View (FPVs) using the multi-criteria decision support methodology of Bana e Costa et al. (1999). This Work Package aims to address the vulnerabilities identified in WP3 by integrating these assets into a comprehensive framework that enhances the managerial effectiveness and resilience of healthcare organizations under the VBHC model. The outcomes of WP4 will serve as a crucial foundation for developing targeted solutions that leverage intangible assets for enhancing the overall effectiveness of the VBHC framework, which are then incorporated into a practical framework in WP5.

For this, a new Systematic Literature Review (SLR) was conducted. Our methodology is inspired by the PRISMA guidelines (Page et al., 2021) but tailored to align with the WBS framework, ensuring a systematic and structured approach to the literature review and subsequent analysis. Although this review was not registered, the protocol outlines the search strategy, inclusion and exclusion criteria, data extraction, and analysis methods. This review was meticulously planned, detailing the search strategy, inclusion and exclusion criteria, and the methods for data extraction and analysis, to ensure a thorough and relevant selection of literature.

Eligibility Criteria: Studies were considered eligible if they discussed intangible assets within organizations, focusing on their identification, measurement, and impact on organizational performance. We included peer-reviewed full articles published in English, Spanish, and Portuguese. Commentary articles, editorials, and conference abstracts were excluded. The time frame for the literature search spanned from 2008 to 2024, chosen due to the significant update in IAS 38 by IFRS on May 22, 2008, which influenced the classification of intangible assets. This change classified new elements as intangible assets, including advertising and promotional activities, production units, and amortization methods [International Financial Reporting Standards (IFRS), 2021].

Given the comprehensive nature of our objective—to detect all intangible assets recognized in scientific literature since 2008—we included a broad range of studies outside the health area to ensure a thorough identification and appropriate clusterization of these assets. This extensive inclusion criteria resulted in the selection of 495 articles, reflecting the diversity and complexity of intangible assets as discussed in the literature.

Information sources and search strategy: a comprehensive search was conducted in the Scopus and Web of Science databases to capture a wide range of discussions on intangible assets. The search strategy commenced with the utilization of the descriptor “intangible assets,” yielding 14,999 studies within the related period. Subsequently, the article selection process involved the combination with the term “measurement,” which resulted in a total of 3,669 scientific articles. It is noteworthy that the juxtaposition of “intangible assets” and “VBHC” (Value-Based Health Care) yielded zero scientific articles, underscoring the urgency of the current reflection proposed. No area filters were applied to ensure a broad capture of relevant studies across various fields.

**Figure 2:**
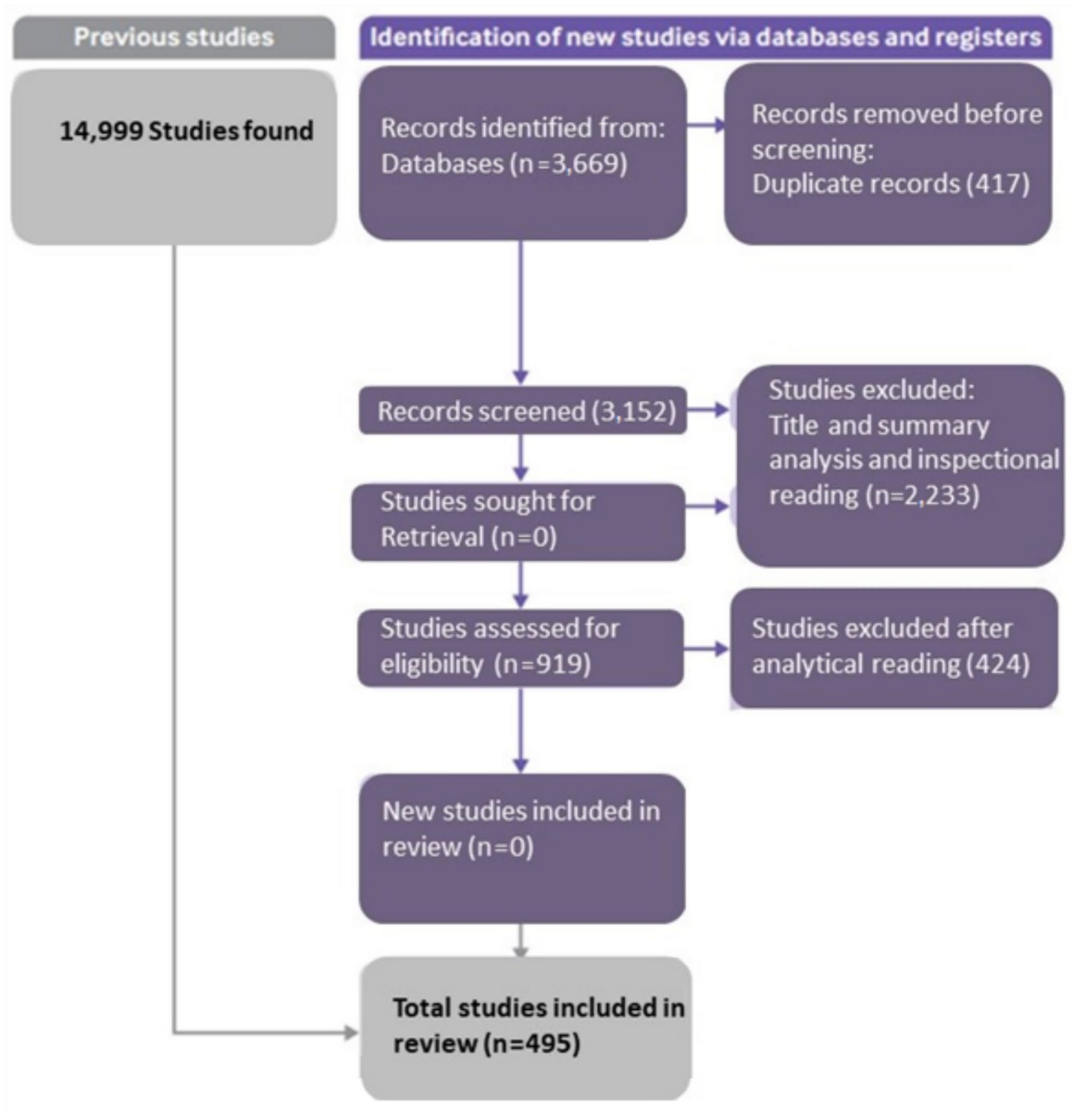
Flow diagram for systematic review (adapted from Page et al., 2021).

Study selection: two reviewers independently screened the titles and abstracts of retrieved records for eligibility. Discrepancies were resolved through discussion or, if necessary, consultation with a third reviewer. Full-text articles were then assessed for inclusion, with reasons for exclusion documented for each excluded study. The primary eligibility criteria in the analytical reading of the articles involved the clarity with which the intangible asset was presented, as the main intention was to obtain data that could contribute to its proper inventory measurement within a healthcare organization. The selection process is summarized in a PRISMA flow diagram (Figure 2).

Data collection process: a standardized data extraction form was used to collect information from each included study. Extracted data included study characteristics (e.g., author, year of publication), methodology, key findings related to intangible assets, and implications for organizational performance and VBHC implementation. The data extraction was performed independently by two reviewers, with discrepancies resolved through consensus, with the intent to categorize intangible assets into FPVs, Critical Success Factors (CSFs), and Indicators, in accordance with the multi-criteria methodology.

Data items: data items of interest included definitions and types of intangible assets identified, methodologies for measuring these assets, and their reported impact on organizational performance within the context of VBHC.

Risk of bias in individual studies: while a formal risk of bias assessment is not typically performed in systematic reviews of non-clinical studies, we assessed the methodological quality of included studies in terms of clarity in defining intangible assets, the rigor of measurement methods, and the strength of evidence linking intangible assets to organizational performance.

Summary measures and synthesis of results: the primary outcome of interest was the identification of intangible assets critical to VBHC implementation. Results were synthesized narratively, highlighting the variety of intangible assets recognized, their measurement, and impact. Where possible, thematic analysis was conducted to categorize intangible assets into broader themes.

Additional analyses: given the heterogeneity of studies, a meta-analysis was not feasible. However, subgroup analyses were planned based on the type of intangible asset to full fill multicriteria analysis. Subgroup analyses were also planned to compare sectors (healthcare vs. non-healthcare); however, this was not feasible as only 16 studies were identified associating intangible assets with healthcare organizations, further highlighting the paucity of discourse within the healthcare field regarding these types of assets.

In response to the PRISMA guideline on “Describe methods used to explore the geometry of the treatment network under study and potential biases related to it,” our study employed a comprehensive multi-criteria methodology, in accordance with Bana e Costa et al. (1999), to systematically analyze and interpret the complex network of treatments. This approach was instrumental in elucidating the intricate interconnections and potential biases within the compiled evidence base. Below, we detail how this methodology aligns with PRISMA requirements:

Introduction to the multi-criteria methodology by Bana e Costa et al. (1999): the segmentation of information into criteria and sub-criteria, as well as indicators measuring the impact of certain activities on a company’s business, represents a nuanced approach. These criteria and sub-criteria can be classified into FPVs, CSFs, and Indicators. An FPV should represent the objectives considered strategic for those making decisions within a company and can form the structure of a decision tree. The CSFs provide a comprehensive approach with a critical focus and clarity of position, indicating which factors will positively impact organizational performance (Venkataraman and Cheng, 2018).

Application of the methodology: we applied the multi-criteria methodology through a structured process involving the identification of FPVs, CSFs, and Indicators. The FPVs in this study were selected to represent the goals considered strategic or primary for the proper recording of intangible assets. This methodology is particularly suited to our study due to its ability to handle diverse data types and its flexibility in accommodating varied research objectives. Each element played a specific role: FPVs provided the foundational analysis framework, CSFs identified essential areas for success to achieve the FPVs, and Indicators offered quantifiable measures of performance. This tripartite structure facilitated a comprehensive evaluation of the evidence base, ensuring a balanced consideration of all relevant factors and was instrumental in the subsequent structuring of the framework.

Exploration of treatment network geometry: to explore the geometry of the treatment network, we utilized the multi-criteria methodology to construct a visual and analytical representation of the evidence network through tables that present them and figures that connect them, subsequently consolidating the presentation of the developed framework.

Identification and mitigation of potential biases: the multi-criteria methodology played a crucial role in identifying and addressing potential biases within the treatment network, particularly by facilitating the exclusion of articles through analytical reading. When a specific FPV, for example, was chosen, the data treatment could be more objective. By systematically evaluating the evidence through these newly predefined criteria, we discerned patterns of bias and implemented corrective measures.

Evidence base compilation and description: the compilation and descriptive presentation of the evidence base were achieved through the meticulous application of our multi-criteria methodology. We presented the key findings, CSFs, and performance Indicators in a manner that is both accessible and informative to readers. This approach ensured that the evidence base is comprehensively described, with clear explanations of how each piece of evidence contributes to the overall analysis.

In addition to our systematic analysis using the multi-criteria methodology, we rigorously assessed the quality of each included study. This evaluation involved a comprehensive examination of study designs, methodologies, and reported outcomes to identify any potential biases or limitations. This critical step ensures that our analysis rests on a foundation of high-quality evidence, further enhancing the reliability and validity of our findings in exploring the treatment network’s geometry.

In conducting this systematic review, we comprehensively identified and assessed the knowledge reported across the included studies. While our review did not perform a meta-analysis due to heterogeneity in study designs, interventions, and reported outcomes, we meticulously categorized and synthesized the findings based on the principal conclusions reported by the studies. By doing so, we aimed to provide a comprehensive overview of knowledge on intangible assets, facilitating informed decision-making and identifying areas for future research. Table 2 resumes the analytical techniques Detailed for WP4 SLR.

**Table 2:** Analytical techniques detailed for WP4 SLR.

Component	Description
Objective	Systematically identify and catalog all intangible assets mentioned in the scientific literature, generating a selection of Fundamental Points of View (FPVs) using the multi-criteria decision support methodology.
Systematic literature review (SLR)	Conducted according to PRISMA guidelines, tailored to align with the WBS framework, ensuring a thorough and structured approach.
Eligibility criteria	Focused on studies discussing intangible assets, their identification, measurement, and impact on organizational performance.
Search strategy and data extraction	Comprehensive search in Scopus and Web of Science; standardized data extraction to ensure consistent information collection.
Multi-criteria decision support	Applied the methodology of Bana e Costa et al. (1999) to categorize intangible assets into FPVs, Critical Success Factors (CSFs), and Indicators.
Thematic analysis	Conducted to identify common themes and patterns, categorizing intangible assets into broader themes.
Exploration of treatment network geometry	Constructed visual and analytical representations of the evidence network.
Identification and mitigation of potential biases	Systematically evaluated evidence to discern patterns of bias and implemented corrective measures.
Synthesis of results	Results synthesized narratively, highlighting the variety of intangible assets recognized, their measurement, and impact.

The specific objective of WP5—Framework Development for Integrating Intangible Assets in VBHC—is to stimulate the creation of a comprehensive framework that inventories intangible assets within healthcare organizations. This framework will integrate both psychological and intangible aspects identified in previous work packages, highlighting their potential to enhance the efficacy and resilience of the VBHC model. The process involves synthesizing the data on intangible assets collected in WP4 and applying innovative design principles to construct a practical and dynamic framework. This framework aims to provide healthcare organizations with actionable strategies to leverage these assets for improved management effectiveness and patient-centered care.

This Work Package will utilize an iterative design process, ensuring that the framework is adaptable and aligns with the evolving needs of healthcare organizations. It will also incorporate feedback from stakeholders to refine the integration of psychological and intangible aspects, thereby maximizing their impact on organizational performance and patient outcomes. The ultimate goal is to develop a model that not only addresses the gaps identified but also sets an updated standard for how VBHC can be implemented more effectively through the strategic identification and use of intangible assets. WP5 utilizes the data and insights from WP4 to develop a tangible framework that can be implemented in healthcare organizations, ensuring the practical application of the research findings.

The specific objective of WP6—Redefining the Value Equation in VBHC: Integrating Intangible Assets through Design Thinking and Open Innovation—is to employ Design Thinking (Brown, 2008) and Open Innovation methodologies (Chesbrough, 2003; Šlapáková Losová and Dvouletý, 2024) to develop an updated exploratory value equation for VBHC, integrating a broader range of intangible assets beyond patient perceptions. This Work Package seeks to utilize the insights and data synthesized from previous WPs to explore innovative solutions that encompass organizational, psychological, and neuroendocrine factors influencing health outcomes. Its principal intention is to update the well-established VBHC value equation (Porter and Teisberg, 2006; Walraven et al., 2021; Riva and Pravettoni, 2016) by incorporating the relevant findings (Gray, 2006; Tsevat and Moriates, 2018; Menear et al., 2019; Fernández et al., 2002) and contributions from this research. By applying Design Thinking, this phase will involve an iterative process of empathy, ideation, prototyping, and testing, aimed at uncovering and addressing complex challenges within healthcare systems. Furthermore, the Open Innovation approach will facilitate collaboration across traditional boundaries, engaging a diverse community of researchers, clinicians, patients, and other stakeholders (Romero and Donaldson, 2023). This inclusive approach ensures that multiple perspectives are considered in the formulation of the updated exploratory value equation, enhancing its relevance and applicability. The aim is to create a value model that not only reflects the clinical outcomes but also values the broader impact of healthcare services on patient well-being and organizational effectiveness. WP6 builds upon the framework developed in WP5, further refining and testing it through iterative processes to ensure its effectiveness and applicability in real-world scenarios.

The specific objective of WP7—Publication of Results—is to thoroughly assess and disseminate the innovation and applicability of the results obtained from the research project using SMART criteria (Specific, Measurable, Achievable, Relevant, Time-bound) as outlined by Drucker (1954). This Work Package aims to compile and publish the findings in a manner that not only showcases the innovative clinical solutions developed but also demonstrates their practical implications and potential benefits in real-world healthcare settings. WP7 ensures that the findings and innovations from all previous WPs are effectively communicated to the broader scientific and healthcare community, promoting further adoption and exploration of the VBHC model enhanced by intangible assets.

In addition to highlighting the innovative aspects of the research, WP7 will critically discuss the methodological limitations and address ethical considerations, particularly concerning the use of human patient data. This discussion will include strategies implemented to ensure the integrity, transparency, and replicability of the study. The ultimate goal is to contribute to the scientific community and healthcare practice by providing a comprehensive overview that encourages further exploration and adoption of the proposed solutions.

Moreover, this phase will involve the meticulous preparation of the results for publication in peer-reviewed journals and presentations at relevant conferences. In the specific preparation of this manuscript, OpenAI’s ChatGPT-4, a generative AI model, was utilized to aid in language refinement and drafting. The model’s responses were carefully reviewed and edited to ensure accuracy and alignment with the authors’ intent. By ensuring that the research findings are communicated effectively and responsibly, WP7 will facilitate the sharing of knowledge and foster a broader understanding and implementation of the enhanced VBHC model developed throughout the project.

## 3 Results

The results illustrate a complex interplay between VBHC, organizational resilience, and intangible assets, suggesting that managerial skills are influenced by factors beyond patient experience and health outcomes, such as intellectual capital and organizational reputation. The integration of data collected in the WPs offers a novel perspective on the synergy between intangible assets and VBHC strategies, indicating updated potential approaches for management. To enhance the clarity and depth of our findings, we further examined the role of each identified intangible asset and its specific impact on VBHC implementation. For optimal clarity, the key findings from WPs 1–4 are presented in Table 3.

**Table 3:** The key methodological points of this study.

WP	Main action(s):	Key points
1	Operationality	A partnership was established with Administration Department of Federal University of Santa Maria. We invited an external collaborators, Luis Felipe Dias Lopes and Claudia de Freitas Michelin, to assist in developing WP4 to WP6.
2	Identifying the basic concepts involved in the study, and the theories involved in understanding the organizational resilience, VBHC and innovation in healthcare.	VBHC (Value-Based Healthcare) is more accessible to a broader audience due to its focus on patient centrality and practical orientation. Its core strength lies in the powerful message that maximizing relevant patient outcomes relative to costs should be the central goal of healthcare (Gray, 2006; Tsevat and Moriates, 2018). VBHC was operationalized by Porter and Teisberg (2006) into six components that were supposed to mutually reinforce each other: organizing care into Integrated Practice Units (IPU’s), measuring outcomes and costs for each patient, shifting to bundled payments for care cycles, integrating care across separate facilities, expanding excellent services across geography, and building an enabling information technology platform.
3	Conducting a integrative analysis of all WP2 data; Understanding the potencial weakness of VBHC.	It is worth noting that the notion of “value” is a central theme in VBHC (Page et al., 2021). According to Walraven et al. (2021), within VBHC, value should always be defined around the customer (patient), not the provider (healthcare or technology provider). When defined from the patient’s perspective, this value concept supposedly encompasses efficiency, safety, patient adherence, and satisfaction. As such, it is inherently multidimensional and can vary among patients and different moments in time. Value is also considered fundamental to achieving other goals such as access and equity. With this perception of value, Porter’s model appears to be in agreement with the definition of health reported by the World Health Organization, where health is a state of complete physical, mental, and social well-being and not merely the absence of disease or infirmity (Riva and Pravettoni, 2016).
4	Combining information from WP2 and WP3 to promote idetification of intangible assets. Choosing crucial success factors for the further advancement of WP5. Confirming the selection of crucial success factors through a comprehensive literature review.	And these gaps around value creation can influence decision-making by generating an “illusion” that all concepts automatically linked to the intangible aspects of a company have ceased to be essential. The proposal to measure value around the focus of patient-centered value creation might allow the emergence of healthcare service structures that undervalue basic management concepts, such as the importance of the intellectual capital of a service-providing company, generating potential biases in strategic planning. And the parameters currently used by VBHC, such as the application of the Learning Health System (LHS), despite potentially optimizing performance and adding to value creation in health systems (Menear et al., 2019), do not incorporate all aspects associated with the value generation of a healthcare company according to Fernández et al. (2002).

As WP3 results, Steinmann et al. (2020), mapping the ambiguity around VBHC, concluded that it is possible to identify four discourses on the perception of the primary purpose of VBHC. Firstly, patient empowerment, strengthening patients’ positions regarding their medical decisions. Secondly, in the governance discourse, VBHC is a toolkit for incentivizing providers. Thirdly, in the professionalism discourse, VBHC is a methodology for the provision of healthcare. Fourthly, in the critique discourse, VBHC is reproached as a manufacturability dogma. Despite divergent lines of reasoning, Steinmann et al. (2020) acknowledged the possibility of shared decision-making as a key component of VBHC.

### 3.1 Segmentation of information into criteria and sub-criteria by WP4

Scientific evidence identified for intangible assets by FPVs represents the strategic goals of an organization and is an important factor in decision-making and determining characteristics of actions taken (Schaefer, 2020). Thus, intangible aspects represent the final level of intangibles for value creation for organizations and depend on the definition of other intangibles identified by the scholarly literature. Table 4 further details the analytical techniques used to identify and categorize these assets, showing the intangible aspects identified by academic review and classified as FPV, as well as providing a visual record of their current correlations with value creation through VBHC. The proposed intangible aspects depend on a strategic level and include other intangibles found by the literature review. FPV represents the main intangible aspects, as they are composed of characteristics present in other intangibles identified by the study (Negreiros et al., 2015).

**Table 4:** Fundamental points of view selected among intangible assets.

FPVs	Definition	Last authors
Performance	Performance describes the contribution of specific systems (organizational units of various sizes, employees, and processes) to achieving and validating a company’s objectives and should consider the quantification of the efficiency and effectiveness of actions.	Liang (2012), Selvam et al. (2020)
Knowledge	Knowledge is a collection of experiences, appropriate information, and skillful insights that offer a framework for estimating and integrating new experiences and information. It is considered a repository of intelligence for the development of organizations.	Ramalho et al. (2020), Ricciotti (2020), Rodgers (2020)
Legitimacy	Legitimacy is a perception or judgment of an organization that society develops. The social support provided to the organization is defined as legitimacy and emerges from an organization’s conformity or congruence with social norms or laws.	Del-Castillo-Feito et al. (2020), Miotto et al. (2020)
Reputation	Corporate reputation can be defined as the collective perception of an organization’s past actions and expectations regarding its future actions, given its efficiency relative to key competitors.	Martins et al. (2021), Baglioni et al. (2021), Arnott et al. (2021)
Innovation	Innovation can be described as a method and technology for new markets, new production methods, and identification of new customer groups. Innovation is an activity where companies solve problems by combining knowledge and can be considered the engine of growth.	Martins et al. (2021), Baglioni et al. (2021), Iriyanto et al. (2021)

**Figure 3:**
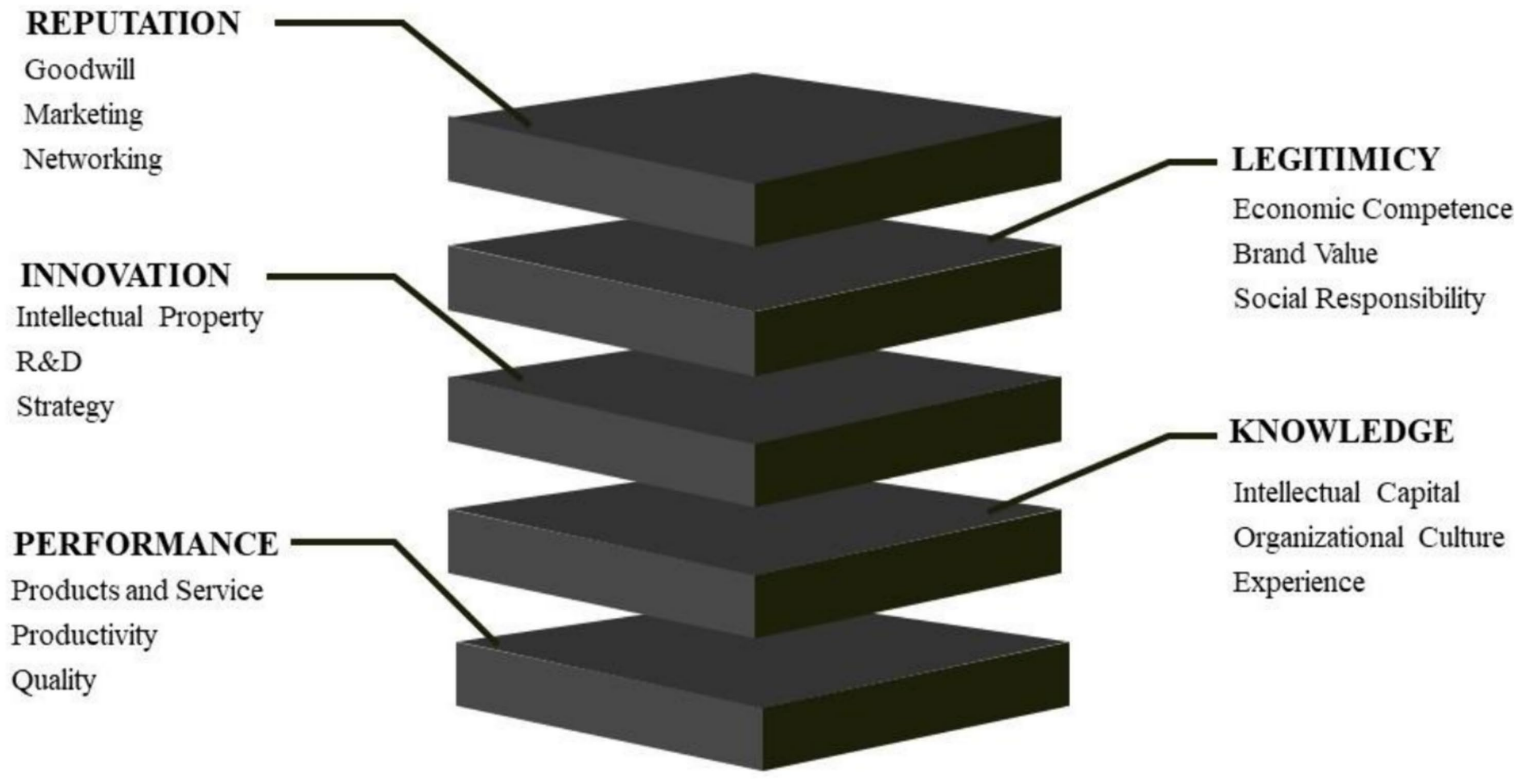
Relationship between each FPV and CSF.

CSFs are the areas in which a company needs to achieve positive outcomes, transforming strategies into concrete actions to reach the proposed objectives (Gupta et al., 2022), and they were also identified. As Wong and Aspinwall (2005) did in their study, CSFs were classified due to the similarity of approaches in relation to FPV. Table 5 presents the intangible aspects identified by the literature review and classified as CSFs, as well as provides a visual record of their current correlations with value creation through VBHC. CSFs are important in assisting the measurement of key viewpoints, with each FPV encompassing a group of CSFs. Figure 3 shows the relationships between each FPV and CSF.

**Table 5:** Intangible aspects identified by the literature review and classified as CSF’s.

CSFs	Definition	Last authors
Goodwill	Goodwill is an asset that represents future economic benefits arising from other assets acquired in a business combination that are not individually identified and recognized separately.	Saastamoinen (2020), Sarfraz et al. (2020)
Marketing	Marketing is an activity, set of institutions, and processes for creating, communicating, delivering, and exchanging offerings that have value for customers, clients, partners, and society at large.	Baglioni et al. (2021), Foroudi et al. (2017), Pastor et al. (2017)
Networking	Business networking can be defined as a process in which many organizations form strong and extensive social, economic, service, and technical ties over time, with the intention of reducing total costs and/or increasing value, thereby obtaining mutual benefits.	Ricciotti (2020), Rodgers (2020), Grimaldi et al. (2017)
Economic competence	Economic competence is the ability to identify, expand, and exploit business opportunities.	Goldar and Parida (2017), Ocak and Findik (2019), Nonnis et al. (2021)
Brand value	Brand value is what exists in the consumer’s mind even in relation to just one aspect of the product: the consumer’s own experience, lifestyle, advice given by friends or opinion leaders, advertising, delivery, ease of use, service availability, warranty, packaging reuse, and much more.	Baglioni et al. (2021), Arnott et al. (2021), Siegrist (2020)
Social responsibility (SR)	Social Responsibility (SR) is a type of international private business self-regulation that aims to contribute to social goals such as philanthropy, activism, or charity by engaging in or supporting volunteering or ethically-oriented practices.	Dodd (2016), Zaragoza-Sáez et al. (2020), Castilla-Polo and Sánchez-Hernández (2020)
Intellectual property	Intellectual property is based on the ability to generate and manage the appropriability of knowledge and the distribution of wealth.	Arnott et al. (2021), Shakina and Barajas (2020), Trappey et al. (2020)
Research and development (R&D)	Research and Development (R&D) comprises the creative and systematic work undertaken to increase the stock of knowledge—including knowledge of humanity, culture, and society—and to devise new applications for available knowledge.	Nonnis et al. (2021), Haskel and Westlake (2021), Garanina et al. (2021)
Strategy	Strategy can also be explained as the ability to choose one or several processes to achieve the organization’s main long-term goals. It also includes courses of action and the allocation of resources necessary to achieve desired objectives.	Del-Castillo-Feito et al. (2020), Zaragoza-Sáez et al. (2020), Rider et al. (2019)
Intellectual capital (IP)	Intellectual Property (IP) is the result of mental processes forming a set of intangible objects that can be used in economic activity and bring income to its owner (organization), encompassing the capabilities of its people, the value relative to its relationships, and everything that remains when employees go home.	Saeidi et al. (2020), Suaedi and Trisliatanto (2020), Wudhikarn et al. (2020)
Organizational culture (OC)	OC is a set of shared assumptions that guide behaviors.	Ramalho et al. (2020), Martins et al. (2021), Baranes (2020)
Experience	Know-how means a wealth of non-patented practical information, derived from the supplier’s experience and testing, which are secret, substantial, and identified.	Podobinska-Staniec and Brzychczy (2018), Sharma and Dharni (2020), García-Gallo (2020)
Products and service	A product is an object or system made available for consumer use; it is anything that can be offered to a market to satisfy the desire or need of a customer. Service is an aggregation of a service commitment with one or more service acts between two or more service systems creating service outcomes.	Igielski (2017), Teniwut and Ngangun (2020)
Productivity	Productivity is the efficiency of producing goods or services expressed by some measure.	Shakina and Barajas (2020), Rider et al. (2019), Trabelsi et al. (2014)
Quality	Quality is a dynamic state associated with products, services, people, processes, and environments that meet or exceed expectations and help produce superior value.	Igielski (2017), Caviggioli et al. (2020), Norliza et al. (2020)

In this study, CSFs were employed to understand and describe intangible aspects that can later be measured through appropriate indicators, commonly associated with intangible assets by the accounting field, which could be utilized to measure the value creation of a venture (Kashirskaya, 2020).

### 3.2 Exploring metrics and references by WP4

Traditional indicators are effective means of supporting decision-making and are focused on organizational efficiency (Chen et al., 2016). Indicators can be defined as metrics that can be used to measure the performance of different objects, tasks, or employees within a company (Ding and Chan, 2013; Kashirskaya, 2020). Table 6 presents the intangible assets identified through literature review and classified as indicators and measurable. The basic characteristic for these intangibles to be classified as indicators was the similarity of concepts with FCS and that metrics could be established for each to be measured.

**Table 6:** Identified indicators for intangible assets.

Indicators	Last authors
Licenses	Siegrist (2020), Baranes (2020), Cerulli et al. (2020)
Relationship with customers	Arnott et al. (2021), Grzes-Buklaho (2018), Tunyi (2020)
Ability	Ramalho et al. (2020), Doran et al. (2020), Gazzola (2020)
Efficiency	Prusak (2017)
Audit	Kashirskaya (2020)
Market research	Haskel and Westlake (2021), Chen et al. (2016), Niebel et al. (2017)
Human capital	Martins et al. (2021), Arnott et al. (2021), Nonnis et al. (2021)
Social capital	Rodgers (2020), Camacho et al. (2020), Sulistyo and Ayuni (2020)
Competence	Foroudi et al. (2017), Eklund (2020), Cortellazzo et al. (2020)
Relational capital	Ramalho et al. (2020), Iriyanto et al. (2021), Mähönen (2020)
Structural capital	Ricciotti (2020), Garanina et al. (2021)
Communication	Graca and Arnaldo (2016), Zuluaga et al. (2017)
Values	Grzes-Buklaho (2018), Guevara and Bounfour (2013), Costa et al. (2014)
Expertise	Nafukho (2009), Madden (2017), Guile and Unwin (2020)
Know-how	Podobinska-Staniec and Brzychczy (2018), Sharma and Dharni (2020), García-Gallo (2020)
Training	Rider et al. (2019), Baldi and Bodmer (2017)
Brand	Baglioni et al. (2021), Arnott et al. (2021), Trappey et al. (2020)
Trademark	Trappey et al. (2020), Baranes (2020)
Mission	Rider et al. (2019)
Social development	Camacho et al. (2020)
Database	Nonnis et al. (2021), Hanafizadeh et al. (2015), Danescu (2020)
Customer list	Pastor et al. (2017), Sprenger et al. (2017)
Alliances	Dodd (2016), Trabelsi et al. (2014), Grzes-Buklaho (2018)
Franchises	Pastor et al. (2017), Baranes (2020)
Relationship with stakeholders (partnership)	Rider et al. (2019), Sharma and Dharni (2020), Durst and Guldenberg (2010), Moriggi (2020)
Image	Del-Castillo-Feito et al. (2020), Graca and Arnaldo (2016)
Advertising	Bontempi and Mairesse (2015)
Business relations	Ricciotti (2020)
Patents	Arnott et al. (2021), Garanina et al. (2021), Tarsalewska (2021)
Copyrights	Baranes (2020), Kossecki and Kossecki (2020)
Software	Arnott et al. (2021), Nonnis et al. (2021), Haskel and Westlake (2021)
Projects	Nonnis et al. (2021), Haskel and Westlake (2021), Edmond and Morselli (2020)
Technologies	Iriyanto et al. (2021), Chen et al. (2016), Neves and Branco (2020)
Business structure	Cho (2020)
Process capital	Grzes-Buklaho (2018), Guevara and Bounfour (2013)

The individual conceptual discrimination of each indicator is beyond the scope of this research stage. However, it is important to clarify that for data to be considered an indicator following international standards, it must have the characteristics of an asset, according to International public sector accounting standards (IPSAS) (2018): (a) it must be identifiable; (b) it must be independent; (c) it must present future economic benefits or service potential; (d) it must have a lifespan (finite or infinite).

### 3.3 WP5—framework for structuring the inventory of intangible assets

The constant need to improve the quality of care, highlighted in reflections inspired by indicators measured by independent entities, such as the Death Quality Index, drives the need for a constant renewal of public health policies (Barber et al., 2017; Flessa and Huebner, 2021). In this scenario, the pressure to innovate in healthcare is a reality (Bauchner et al., 2016; Yamey and Morel, 2016). Innovation in healthcare goes beyond simple technological advancements, as it must also promote evolution in basic science as well as healthcare funding systems, as otherwise there will be no impact on patients’ lives (Bauchner et al., 2016; Henderson et al., 2000). Consistent innovation should encompass both the new product itself and innovation in procedures, as well as allow for the proper documenting of information that can theoretically serve as the basis for, as well direct its subsequent implementation in public policy (Schaefer, 2020; Yamey and Morel, 2016).

Amid this push for innovation, the conception of a framework emerges not just as a response but as a necessity, bridging the gap between evolving healthcare practices and the structured assessment of their value. This framework is designed to harness this momentum, providing essential technical guidance for the systematic collection and valuation of intangible assets, which are increasingly recognized as pivotal in driving quality and sustainable growth in healthcare (Dal et al., 2020; Jelonek and Halilovic, 2016).

The development of a framework aims to provide technical guidance for the collection of specific data on each intangible aspect for the future preparation of financial statements, in order to enable value creation for the organization. The scope of this structure offers guidance for collecting data to assist in the recognition and measurement of intangible aspects of exploration and evaluation. To clarify clustering and identification, FVPs and CSFs are considered intangible aspects, and Indicators are considered intangible assets, following this logic: each FVP represents the main topic for information collection, systematically generating five sets of independent data.

When observing the indicators for the proper evaluation of each criterion, it is noticeable that for the adequate creation of value of a healthcare company, even though VBHC logistics, information beyond the experience registered with the patient is necessary. Value creation is the appropriation and return on investment obtained by companies in compensation for their value propositions and offerings (Teece, 2010). Value creation arises through new combinations of resources to create new products, services, or new production methods (Oliveira et al., 2021).

The academic literature provides a range of intangibles that influence the value creation of companies and also provides evidence that these intangibles can be classified and managed. Thus, the opportunity to study how to identify and structure these intangibles arises. Therefore, the aim of this article is to present a framework that identifies the true intangible aspects that contribute to value creation for organizations, classified according to their respective FVP’s, CSF’s, and Indicators.

**Figure 4:**
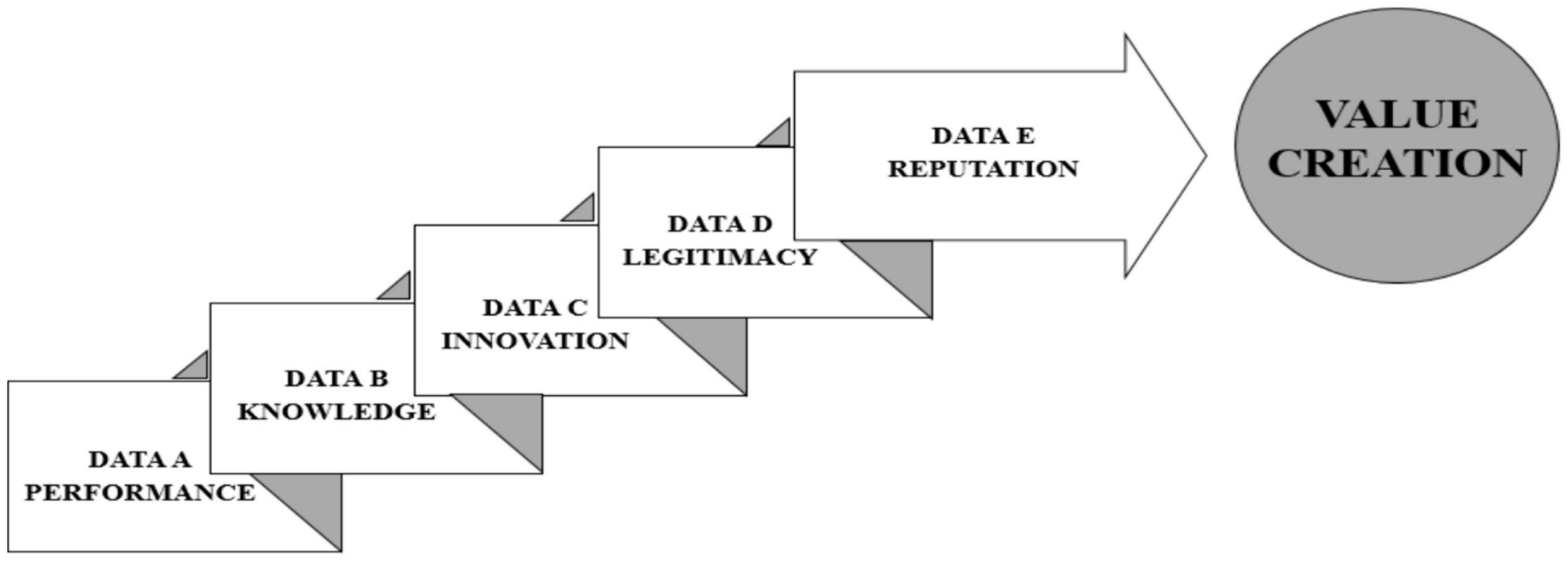
Analysis of the value creation steps from intangible assets.

The framework presents a set of intangibles introduced by the literature over the years and can serve to guide new studies, decisions, and actions of companies regarding intangibles. Figure 4 shows the process of value creation according to the specific data generated by each of the five FVPs’.

**Figure 5:**
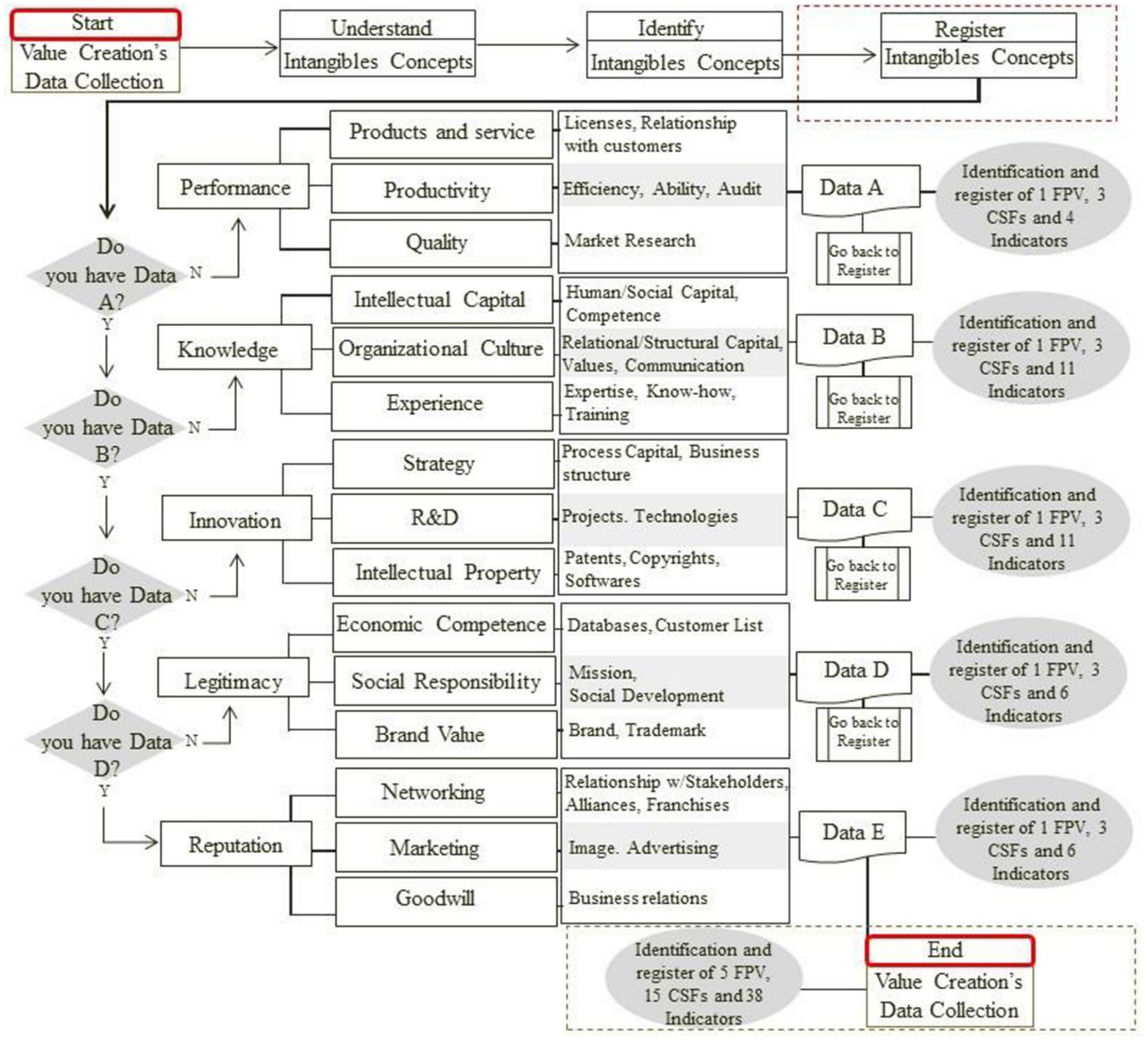
Framework of steps for data capture to collect intangible assets in healthcare companies.

The data collection sequence follows an order so that all data is correctly collected. Initially, data related to FPV Performance is collected through information associated with directly related FCSs to Products and Services, Productivity, and Quality. These, in turn, through their respective indicators, will provide the information termed Data A. After this data is collected, the flowchart is repeated for data collection related to the FPV called Knowledge, creating the information dataset named Data B. The process is repeated for the remaining FPVs. Thus, after collecting Data C, Data D, and Data E, the information for the company’s value creation is complete. Figure 5 shows the preliminary Theoretical Framework for data capture to collect intangible assets in healthcare companies.

Some specific rules based on the International public sector accounting standards (IPSAS) (2018) standards must be followed so that the identified FCSs and indicators can be subsequently measured. These rules are described below:

- Internally generated datum: an internally generated FCS datum should not be recognized as an indicator because it is not an identifiable resource controlled by an entity that can be reliably measured and does not come from a binding agreement. For example, Goodwill, which can be identified as part of the company’s organizational culture.
- Acquisitions: indicators acquired separately (for example, a license agreement, etc.) should be independently recognized and recorded with the data related to their corresponding FCSs, creating a specific dataset for each FPV.
- Internally generated intangible indicators may present recognition problems due to the difficulty of identifying whether the indicator creates economic benefits or because it reliably determines asset cost. Thus, for an internally created intangible asset to meet recognition criteria, it is possible to classify the research phase and the development phase.
- To determine whether an intangible indicator, whether with a defined or indefinite lifespan, is impaired, it must be documented.
- An intangible indicator must be unknown, if there is disposal (including disposal through a transaction without consideration); or when no future economic benefit or service potential is expected from its use or disposal (losing the asset characteristics).

Upon encountering a framework that systematizes the detection and evaluation of intangible assets, a healthcare organization manager can gain various advantages, listed in Table 7. For a manager operating under the principles of VBHC, the implementation of a framework focused on intangible assets can offer significant complementary benefits, aiding in value focus. This is because VBHC emphasizes the importance of maximizing value for patients. Intangible assets, such as patient satisfaction and accumulated clinical knowledge, are central elements in delivering value. By quantifying and managing these assets, the manager can boost health outcomes and patient experience, aligning even more closely with the objectives of VBHC.

**Table 7:** Advantages for a healthcare company manager gained through a framework that systematizes the detection and evaluation of intangible assets.

Advantages	Description
Integrated asset view	The manager gains a clear and structured view of the intangible assets, often not accounted for, but crucial for the organization’s long-term success, such as organizational culture, patient satisfaction, and intellectual capital.
Informed Decision-Making	With a better understanding of intangible resources, the manager can make more informed decisions on where to invest in improvements, whether in training, technology, or management practices.
Strategic alignment	Using the framework, the manager can align the intangible assets with the organizational strategy, ensuring they effectively contribute to the VBHC objectives, such as improving healthcare quality and service efficiency.
Opportunity identification	The framework can help identify underutilized intangible assets or areas for improvement that could become competitive advantages, such as innovative training programs or a reputation for excellence in patient care.
Continuous improvement	By highlighting the importance of intangible assets, the manager can promote a culture of continuous improvement, fostering innovation and employee commitment.
Brand and reputation enhancement	Recognizing and developing assets like brand and reputation, the company can strengthen its market position, which can translate into greater patient loyalty and better strategic positioning.
Long-term financial impact	Strong intangible assets can lead to better financial performance over time through patient retention, cost reduction, and enhanced operational efficiency.
Competitiveness and innovation	By valuing assets such as knowledge and intellectual property, the manager can boost the organization’s competitiveness and innovation capacity.
Social responsibility and governance	A deeper understanding of intangible assets can promote social responsibility and good governance practices, aligning the company with best practices and stakeholder expectations.
Future preparedness	In a rapidly transforming sector like healthcare, having a framework for intangible assets prepares the organization to adapt and thrive in the face of regulatory, technological, and market changes.

Furthermore, the framework allows for the systematic collection and analysis of data on intangible assets, providing a solid foundation for evidence-based decisions that are in tune with VBHC recommended practices. By focusing on intangible assets, such as the efficiency of internal processes or innovation culture, the manager can differentiate their organization in an increasingly value-oriented market. This promotes their competitive differentiation, financial sustainability, and improvements in care coordination. At the same time, attention to intangible assets, like organizational culture and employee engagement, can lead to greater alignment with VBHC values, promoting patient-centered care and a collaborative work environment.

The framework can also foster innovation in care models, encouraging managers to promote adaptation and resilience. The COVID-19 pandemic has underscored the importance of having adaptable and resilient healthcare systems (Shahzad et al., 2023). The management of intangible assets is crucial for resilience, as it encompasses the capacity for innovation and swift response to changes. Moreover, new performance measurement metrics can be introduced. A framework that includes intangible assets enables the measurement of performance in ways that traditional financial and clinical indicators do not capture, such as innovation and patient satisfaction. Therefore, adopting a framework that integrates the identification and management of intangible assets represents an advanced strategy for healthcare managers aiming to maximize the value of patient care and improve organizational performance within the VBHC model. In summary, adopting a framework for the management of intangible assets can transform how managers understand and operate their healthcare organizations, leading to significant improvements in both the quality of patient care and the organization’s sustainability.

### 3.4 WP6 results—the updated exploratory value equation

In this context, an updated exploratory equation for VBHC (Value-Based Healthcare) is proposed, reflecting a broader understanding of value that transcends direct clinical outcomes. It includes factors that significantly contribute to the patient’s overall experience and well-being, as well as organizational efficacy. While the traditional VBHC equation predominantly focuses on health outcomes relative to cost, this updated equation integrates the intangible assets that impact both outcomes and organizational sustainability. By incorporating insights from this study, the equation is reformulated to better capture the comprehensive and long-term value within healthcare systems. The proposed updated exploratory equation for VBHC is as follows:


$$\text{Value}\;\left(\text{VBHC}\right)=\frac{\text{Healt}\;\text{Outcomes}+\text{Intangibles}\;\text{Benefits}}{\text{Total}\;\text{Cost}}$$

where:

- Health Outcomes: Continue to be a central measure, including clinical efficacy, quality of life, and patient satisfaction;
- Intangibles Benefits: A new component that encompasses variables such as Intellectual Capital (accumulated knowledge and skills contributing to innovation and therapeutic efficacy); Social Capital (relationships and networks within and outside the organization that facilitate efficient care coordination); Organizational Reputation (external perceptions of quality and reliability that can influence patient choices and partnerships); Organizational Culture (factors such as employee engagement, work ethics, and alignment with health missions impacting motivation and productivity); and, Social Responsibility (contributions of the organization to the community and sustainable practices that enhance public acceptance and support);
- Total Cost: Includes all costs associated with care delivery, not limited to direct treatments, administration, and capital investments.

This updated exploratory equation acknowledges that value in VBHC is not merely a function of clinical outcomes relative to cost, but also includes the benefits brought by these intangible assets. This reflects a more comprehensive and sustainable view of healthcare, aligning more closely with patient needs and expectations, while enhancing organizational management. Table 8 reviews the key points.

**Table 8:** The key points review.

Topic	Description
Integration of outcomes and intangible assets:	The explicit inclusion of intangible assets in the value equation underscores the importance of considering how elements like knowledge, culture, and reputation can directly and indirectly impact health outcomes and organizational efficiency.
Practical application:	This equation enables healthcare managers to more effectively measure and manage resources that create long-term value, not only through clinical outcomes but also through the more effective management of intangible resources.
Strategic implications:	With this approach, healthcare organizations can differentiate themselves in a competitive market, not only through their clinical capabilities but also through social capital, innovation, and social responsibility.

This updated formulation could serve as a valuable starting point for the discussion and implementation of VBHC practices that fully recognize and utilize intangible assets, promoting continuous improvement in both healthcare delivery and organizational management.

Measuring intangible assets presents a significant challenge, particularly because they often do not lend themselves to direct quantitative assessments like tangible assets. However, developing effective metrics for intangible assets is crucial for their successful integration into the updated value equation in VBHC. Table 9 offers some suggestions for developing metrics for each identified intangible asset, including specific indicators that can reflect its contribution to organizational objectives and health outcomes.

**Table 9:** Suggestions for developing metrics for each identified intangible asset.

Intangible Asset	Suggestions for developing metrics
Intellectual capital	Measured through indicators such as the number of innovations implemented, scientific publications, and rates of adoption of new evidence-based practices.
Social capital	Assessed through metrics such as the strength of the organization’s partnership network, frequency, and quality of interactions with other healthcare institutions, and partner satisfaction.
Organizational reputation	Evaluated using external perception surveys, healthcare publication rankings, and social media analytics.
Organizational culture	Measured by employee engagement and satisfaction surveys, turnover rates, and feedback on employee satisfaction.
Social responsibility	Gauged by community engagement measures such as the number of outreach programs, social impact assessments, and recognitions for sustainable practices.

These approaches not only aid in more effectively quantifying intangible assets but also facilitate their integration into daily operations and long-term strategy of healthcare organizations. The key is to ensure that these metrics are consistently evaluated and adjusted as necessary to accurately reflect the real value that intangible assets bring to the organization and its patients. Incorporating intangible assets into the value equation in VBHC in a structured and measurable way can open doors to significant improvements in healthcare management and delivery. This not only can lead to a more comprehensive understanding of how intangible aspects influence health outcomes but also promotes a more holistic and patient-centered approach to healthcare. The ability to quantify and integrate these assets could be a major breakthrough, allowing healthcare organizations to not only improve clinical outcomes but also enrich the patient experience and strengthen organizational efficacy. If implemented correctly, this approach could become an influential model that others might follow, contributing to the transformation of healthcare practices globally.

### 3.5 WP7 results—interplay synthesis between VBHC, organizational resilience, and intangible assets

The results illustrate a complex interplay between VBHC, organizational resilience, and intangible assets, suggesting that managerial skills are influenced by factors beyond patient experience and health outcomes, such as intellectual capital and organizational reputation. The integration of data collected in the WPs offers a novel perspective on the synergy between intangible assets and VBHC strategies, indicating new potential approaches for management.

The WP4 SLR utilized a comprehensive, multi-criteria decision support methodology to classify intangible assets into Fundamental Points of View (FPVs), Critical Success Factors (CSFs), and Indicators. This structured and detailed evaluation focused on identifying and integrating intangible assets critical for enhancing the VBHC framework. The thematic analysis identified common patterns and correlations between intangible assets and VBHC outcomes, facilitating the development of a practical framework in WP5.

The results were assessed and disseminated using SMART criteria (Specific, Measurable, Achievable, Relevant, Time-bound), ensuring that the innovations and their practical implications were clearly demonstrated (Table 10). This strategic approach enabled the development of actionable insights and targeted solutions to enhance VBHC frameworks, focusing on both immediate and long-term impacts.

**Table 10:** SMART Criteria for WP7.

Criteria	Description
Specific	Clearly define the updated exploratory value equation for VBHC, incorporating intangible assets such as intellectual capital, organizational culture, and reputation. Specify the key intangible assets to be measured and their respective roles in enhancing VBHC strategies.
Measurable	Develop specific indicators for each intangible asset identified, such as metrics for patient satisfaction, clinical knowledge, and organizational culture. Implement measurement tools and methodologies to assess the impact of intangible assets on health outcomes and organizational performance.
Achievable	Ensure the proposed strategies for integrating intangible assets into VBHC are practical and feasible within the existing healthcare framework. Leverage existing resources and infrastructure to implement the updated exploratory value equation effectively.
Relevant	Align the integration of intangible assets with the overall goals of VBHC, focusing on improving patient outcomes, organizational resilience, and healthcare quality. Highlight the importance of intangible assets in driving innovation and value creation in healthcare settings.
Time-bound	Set clear timelines for the implementation and evaluation of the updated exploratory value equation, including short-term and long-term milestones. Establish periodic reviews to assess progress and make necessary adjustments to the strategies.

The study highlights the interconnectedness of VBHC, organizational resilience, and intangible assets, providing a deeper understanding of their combined impact on healthcare management. By systematically identifying and integrating intangible assets, the research presents a refined approach to VBHC that emphasizes the importance of intellectual capital, organizational culture, and social capital in driving value creation and resilience in healthcare organizations. This new synthesis offers a robust framework for future research and practical application in healthcare management.

The importance of identifying intangible assets such as intellectual capital and organizational reputation is critical for optimizing VBHC strategies. By systematically cataloging these assets, WP4 has provided a foundation that highlights how intangible assets contribute to managerial effectiveness and organizational resilience.

This research underscores the interplay between organizational resilience and intangible assets, such as human capital, and identifies their crucial role in the effective implementation of VBHC, providing additional theoretical support. It is well established that organizational resilience is directly linked to the intangible characteristics that a healthcare institution possesses. For example, the relationship between social determinants of health and resilience has been investigated at the individual level and, to some extent, at the community level. The aftermath of the COVID-19 pandemic further highlighted the necessity for organizational resilience in the United States. The US public health and healthcare system began the lengthy process of identifying the resiliency needs of its workforce that expand beyond disaster preparedness (Cook and Stewart, 2023). Organizational resilience is associated with perceived well-being and employee resilience, which are crucial for the overall effectiveness of VBHC implementations (Wut et al., 2022). Additionally, Shepherd and Williams (2023) provided new insights into different paths to resilience based on differences in how organizations interpret and respond to adverse events. Their study offers a model of organizational response paths to resilience, complementing the notion of post-adversity growth by explaining how organizations grow during adversity. This perspective enhances the understanding of the dynamic and multifaceted nature of organizational resilience, particularly in the context of environmental shocks like the COVID-19 crisis. Furthermore, Wilson (2016) highlights the central importance of expanding decision-making boundaries in the resilience of organizations and their ability to adapt under adverse conditions, such as bankruptcy. This supports the development of a human resource strategy to build organizational resilience, which is essential for navigating crises and ensuring the sustained success of VBHC.

## 4 Discussion

The results of the applied research highlight the critical role of intangible assets in optimizing VBHC, revealing a gap in the integration of these assets within healthcare organizations. Our findings align with recent literature emphasizing the importance of intangible assets in healthcare management (Kidanemariam et al., 2023; van der Voorden et al., 2023; Bandurska et al., 2023). One limitation of this study includes potential selection bias towards studies available in the searched databases and published in the specified languages. Additionally, the exclusion of grey literature and potential publication bias may affect the comprehensiveness of our analysis. Future research should expand the search strategy to encompass more diverse sources and potentially unpublished studies to mitigate these limitations.

Clinical outcomes refer to changes in patients’ health conditions resulting from medical interventions or treatments. These include objective measures such as survival rates and readmissions, as well as subjective measures like quality of life and patient satisfaction. In VBHC, clinical outcomes are crucial for assessing the value provided by a particular treatment or intervention. A precise and comprehensive assessment of these outcomes allows for a more complete analysis of the true impact of medical practices on patient health. As demonstrated by Santos et al. (2021), the pandemic has made this process of change even more important. The incentives behind the adoption of Value-Based Health Care (VBHC) contribute to the reduction of waste, focus of the care model on the patient, and integration of the health system.

In Brazil, VBHC has evolved from processes focused on the care line to outcome-based agreements, such as shared risks and pay-for-performance. It is important to highlight that in this sector, success stories concerning value-based models in commercial negotiations still have low visibility. The initial focus on improving clinical outcomes in care lines results in fewer complications, shorter hospital stays, and greater patient satisfaction (Makdisse et al., 2020).

Transitioning from the current system to a value-based one is not a simple task and requires more than introducing incremental improvements, such as evidence-based protocols, Lean programs for continuous process improvement (Yadav et al., 2017), management of high-risk patients, and measures to discourage resource use, such as prior authorization and copayment. Although these approaches are important, by themselves, they do not have the power to drive a complete system transformation and ensure the delivery of health outcomes. However, it is relevant to mention that many of these incremental improvements represent valuable tools in the implementation of Value-Based Health Care (VBHC).

Measuring complex clinical outcomes, such as quality of life, can be challenging. VBHC faces the need to develop robust and sensitive methods to capture subjective and multifaceted aspects of health outcomes (Steinmann et al., 2020). The risk of strategically directing measurement of value while prioritizing the end result, as proposed by VBHC, can create a potential administrative bias, complicating its implementation.

Steinmann et al. (2020) identified four talking points presenting distinct interpretations of VBHC’s primary goal. Firstly, patient empowerment, in which VBHC is a framework to strengthen patients’ positions regarding their medical decisions. Secondly, governance, in which VBHC is a toolkit for incentivizing providers. Thirdly, within discussions about professionalism, VBHC is a methodology for the provision of healthcare. Fourthly, among critics, VBHC is reproached as a manufacturability dogma. Despite these divergent lines of reasoning, there is a common understanding: the perception that shared decision-making is a key component of VBHC.

In 2013, Porter and Lee published a framework to aid in the implementation process known as the Value Agenda. It is comprised of six interrelated elements: organizing into integrated practice units; measurement of health outcomes and costs for every patient; bundled payments for care cycles; care integration across health services; geographic expansion of excellent services; and the provision of an Information Technology platform that supports the value strategy (Porter, 2010).

Although Porter does not use the term “intangible assets” in his theory, several factors he described as essential are indeed considered as such by the scientific literature on the subject. In summation, VBHC advocates for a shift to a more coherent health system, composed of six interdependent elements: (a) organizing care into integrated practice units; (b) measurement of outcomes and costs for each patient; (c) reimbursement through bundled payments for complete care cycles (from start to final stage); (d) provision of integrated care; (e) geographical expansion of services with the best outcomes; and (f) creation of facilitating information technology platforms. All these create intangibles, including organizational culture and intellectual capital (Porter, 2010; Makdisse et al., 2020).

Recently, Kidanemariam et al. (2023), van der Voorden et al. (2023), and Bandurska et al. (2023) pointed out that the VBHC strategy, despite aiding in understanding the healthcare sector, structuring processes, and optimizing financial management, complicates its own implementation by failing to recognize how to properly identify various crucial intangible assets within its logistics. Kidanemariam et al. (2023) exposed a knowledge gap in VBHC by showing that evidence on this methodology supporting patient-centered care is limited. They demonstrated that the measures most frequently used in VBHC research are not patient-centered.

The main focus seems to be on quality of care measures defined from the perspective of a provider, institution, or payer. The difficulty of measuring this intangible asset, consumer satisfaction, appears to be the main reason. In line with this thought, van der Voorden et al. (2023) adds that one of the key elements of VBHC is a clear understanding of which outcomes are most important to patients. Bandurska et al. (2023) describe that the greatest difficulties related to the implementation of the VBHC concept are the lack of legal and reimbursement solutions, personnel shortages, lack of educational standards for some members of the multidisciplinary team, and insufficient awareness of the role that integrated care plays. These last three involve Human and Intellectual Capital, known intangible assets.

Thus, knowing how to identify intangible assets has been considered fundamental for the proper inventory of a healthcare company and appears to be the main and immediate point of difficulty behind implementing VBHC. It is essential for healthcare managers to recognize that intangible assets contribute to a deeper understanding of health outcomes and the quality of care, and therefore, that these are essential for a successful implementation of VBHC and expanding healthcare system improvements. Moreover, several recent studies directly associate intangible assets with value creation in companies from other sectors.

Future research should explore the development of robust and sensitive methods to capture subjective and multifaceted aspects of health outcomes in VBHC. Additionally, there is a need for studies that further investigate the integration of intangible assets in healthcare management, particularly in different cultural and organizational contexts.

The limited number of studies identified associating intangible assets with healthcare organizations points to an underdeveloped area in healthcare research, suggesting a need for further exploration. This systematic review contributes to the existing literature by not only identifying but also categorizing crucial intangible assets, thereby providing actionable insights for healthcare managers to integrate these assets into their strategies.

Our WP2 and WP4 reviews faced limitations, including potential selection bias towards studies available in the searched databases and published in the specified languages. The exclusion of grey literature and the potential publication bias could also affect the comprehensiveness of our WP2 and WP4 analysis. However, the creative process conducted in WP3, WP5, and WP6 provided conclusions that are robust and well-supported. Future reviews could expand the search strategy to encompass more diverse sources and potentially unpublished studies to mitigate these limitations.

The findings from our review imply that healthcare managers should prioritize the identification and measurement of intangible assets as a strategic imperative. These findings support the implementation of VBHC by emphasizing the value of assets like patient satisfaction and organizational culture, which have been less acknowledged in the transition from traditional healthcare models.

Moreover, organizational resilience is a critical aspect revealed through our analysis. The ability of healthcare organizations to adapt, recover, and thrive amid crises, such as the COVID-19 pandemic, highlights the importance of intangible assets in fostering resilience. Organizational resilience refers to the capacity of an organization to anticipate, prepare for, respond to, and adapt to incremental change and sudden disruptions to survive and prosper. In the context of VBHC, resilience is underpinned by robust intangible assets, such as intellectual capital, organizational culture, and social capital, which collectively enhance the ability of healthcare organizations to withstand and adapt to challenges.

Kidanemariam et al. (2023) highlighted that the resilience of healthcare systems during the COVID-19 pandemic was significantly influenced by the presence and effective management of intangible assets. These assets enabled organizations to swiftly adapt to new care protocols, ensure continuity of care, and maintain operational efficiency under unprecedented strain. Thus, integrating intangible assets into the strategic framework of VBHC not only enhances the quality of care and patient satisfaction but also strengthens the overall resilience of healthcare organizations.

## 5 Final remarks and implications

Measuring the value of intangible assets, such as company culture, knowledge management systems, and employee skills, presents a significant challenge for the accounting sector. Executives understand that these intangibles, due to their unique nature and difficulty to imitate, serve as powerful sources of sustainable competitive advantage. Facilitating their measurement will enable more precise and easier control of the company’s competitive position.

The current theoretical framework provides an extensive overview of existing research involving intangibles. Through a systematic review, it was possible to establish a classification and summary of intangibles according to different levels, always with the ultimate goal of value creation for companies. No domain filters were added to database searches to ensure that all intangibles present in the literature were included and made part of a set of best practices, which can be measured and applied to any domain.

The intent is to reaffirm that performance in healthcare service provision means more than just measuring care outcomes. It is essential to emphasize how healthcare organizations learn to utilize all this measurement information in decision-making. The theoretical implication of this paper is that the set of intangibles has been classified into a structure that begins with indicators, identified as intangible assets in the scientific literature, which formulated the CSFs, which, in turn, complete the FPVs.

From the previous context, this study focuses on optimizing the VBHC concept based on the use of intangible assets, considering their growing importance, such as knowledge, intellectual property, and human capital, in the proper provision of healthcare services. The stimulus for this inclusion is driven by the pressure for innovation in healthcare and the constant need for increased efficiency and effectiveness of services provided by healthcare institutions.

By considering these indirect intangible assets, healthcare organizations can make appropriate decisions to maximize the value they generate for their patients, which is the essence of the VBHC theory. However, these intangible assets are just some of the possible ones to be identified in a company. Proper identification of them will expand value creation, even if VBHC remains the main management tool used.

Focusing solely on the end result (the positive impact measured in one way or another on the patient) unfortunately has the potential to create an administrative bias, as important intangible assets, such as an organization’s intellectual capital, might be overlooked. Moreover, the constant identification of intangible assets in healthcare organizations has been considered fundamental for the proper inventory of a company. Therefore, while the VBHC strategy may optimize financial management, by ignoring various crucial intangible assets, it may weaken the overall value generation of a company. Emphasizing this concern, Demers et al. (2021) stressed that governance alone was insufficient for managing the health crisis generated by COVID-19, but investments in intangible assets were crucial.

Furthermore, service provision presents other challenges beyond administrative ones. Reflections inspired by indicators measured by independent entities, such as the Death Quality Index (Barber et al., 2017), highlight the need for constant renewal of public policies to improve the quality of healthcare offered to the population, whether related to innovations in healthcare or not. All this know-how is an essential intangible asset that should always be encouraged by health organizations. Successful healthcare systems will have the means to innovate in service provision that transcends organizational, political, geographical, and sectoral boundaries. Although these concepts are not new, robust and easily accessible practices and structures for their effective integration into daily operations and culture of health systems are still limited. Thus, recognizing the need to innovate creates a reflection on administrative strategies, highlighting the importance of adding the concept of intangible assets to the VBHC management tool.

It is expected that this inclusion can increase the competitiveness and sustainability of health organizations, enabling the consistent achievement of performance goals and promoting cooperation and coordination. It should be emphasized that business competitiveness in the context of health institutions refers to the continuous search for best practices, innovation, operational efficiency, and quality of care to stand out in the market and provide superior healthcare to patients, since the characteristics of many health systems often inhibit the degree to which there can be market competition, i.e., with providers competing for patients.

Stimulating the observation, identification, and encouragement of intangible assets in health institutions can bring significant contributions to the inventory elaboration of a company, in addition to directly stimulating innovation in healthcare. Moreover, innovative approaches and the constant search for advanced solutions in an institution stimulate the organizational culture of innovation, encouraging intangible values such as creativity, scientific curiosity, and the pursuit of best practices.

Furthermore, prioritizing intangible values strengthens the doctor-patient relationship. Empathy and trust are fundamental for building a strong bond between the healthcare team and the patient, increasing adherence to treatment and promoting open and collaborative communication. Valuing intangible assets also impacts the quality of healthcare provided. Ethics, humanism, and commitment to clinical excellence positively influence healthcare quality, resulting in better clinical outcomes, reduction of medical errors, and greater patient safety.

Regarding practical implications, this research may assist health managers in recognizing the importance of intangible assets and standardizing data collection to create value for organizations from the intangible aspects presented in the scientific literature, without neglecting the importance of value measurement through the VBHC methodology. The results of the study will contribute to the management of intangible aspects, from aiding in investment planning to the perception of business competitiveness. These agents could develop work activities to improve indicators in health companies. The assimilation of steps in the theoretical framework is advantageous for understanding the order and arrangement of activities that make up this process. Thus, this stage of the work can base health managers’ perception of the effects generated by the management of intangibles.

Future research should explore how different cultural and organizational contexts impact the integration and management of intangible assets in VBHC. Additionally, developing methodologies to measure the impact of these assets more accurately will be crucial for further advancements in this field.

Our applied research underscores the imperative of acknowledging and integrating intangible assets in healthcare management as essential to the true realization of VBHC. This integration not only has the potential to enhance the quality of patient care but also serves as a cornerstone for the strategic development of healthcare organizations in an increasingly value-oriented industry.

In conclusion, recognizing and effectively managing intangible assets are paramount for the successful implementation of VBHC. These assets play a vital role in enhancing organizational resilience, optimizing healthcare delivery, and improving patient outcomes. Therefore, healthcare managers should strategically integrate intangible assets into their organizational practices, ensuring a more comprehensive and resilient approach to healthcare management. This integration will facilitate the transition towards a more value-based, patient-centered, and resilient healthcare system, ultimately contributing to the sustainability and effectiveness of healthcare organizations globally. The findings from our applied research imply that healthcare managers should prioritize the identification and measurement of intangible assets as a strategic imperative. These findings support the implementation of VBHC by emphasizing the value of assets like patient satisfaction and organizational culture, which have been less acknowledged in the transition from traditional healthcare models.
